# Distribution and regulation of gonadotropin-releasing hormone, kisspeptin, RF-amide related peptide-3, and dynorphin in the bovine hypothalamus

**DOI:** 10.7717/peerj.1833

**Published:** 2016-03-21

**Authors:** Valeria M. Tanco, Brian K. Whitlock, Melaney A. Jones, Robyn R. Wilborn, Terry D. Brandebourg, Chad D. Foradori

**Affiliations:** 1 Large Animal Clinical Sciences, College of Veterinary Medicine, University of Tennessee—Knoxville, Knoxville, TN, United States; 2 Department of Anatomy, Physiology, and Pharmacology, College of Veterinary Medicine, Auburn University, Auburn, AL, United States; 3 Department of Clinical Sciences, College of Veterinary Medicine, Auburn University, Auburn, AL, United States; 4 Department of Animal Sciences, College of Agriculture, Auburn University, Auburn, AL, United States

**Keywords:** Endogenous opioid, Cattle, GnIH, Gonadotropin-inhibitory hormone

## Abstract

Recent work has led to the hypothesis that kisspeptin/neurokinin B/dynorphin (KNDy) neurons in the arcuate nucleus (ARC) play a key role in gonadotropin-releasing hormone (GnRH) pulse generation and gonadal steroid feedback, with kisspeptin driving GnRH release and neurokinin B and dynorphin acting as pulse start and stop signals, respectively. A separate cell group, expressing RFamide-related peptide-3 (RFRP-3) has been shown to be a primary inhibitor of GnRH release. Very little is known regarding these cell groups in the bovine. In this study, we examined the relative immunoreactivity of kisspeptin, dynorphin, and RFRP-3 and their possible connectivity to GnRH neurons in the hypothalami of periestrus and diestrus bovine. While GnRH and RFRP-3 immunoreactivity were unchanged, kisspeptin and dynorphin immunoreactivity levels varied in relation to plasma progesterone concentrations and estrous status. Animals with higher plasma progesterone concentrations in diestrus had lower kisspeptin and increased dynorphin immunoreactivity in the ARC. The percentage of GnRH cells with kisspeptin or RFRP-3 fibers in close apposition did not differ between estrous stages. However, the proportions of GnRH cells with kisspeptin or RFRP-3 contacts (∼49.8% and ∼31.3%, respectively) suggest direct communication between kisspeptin and RFRP-3 cells to GnRH cells in the bovine. The data produced in this work support roles for kisspeptin and dynorphin, within the KNDy neural network, in controlling GnRH release over the ovarian cycle and conveying progesterone-negative feedback onto GnRH neurons in the bovine.

## Introduction

The release of the decapeptide gonadotropin-releasing hormone (GnRH) into the portal vascular system is the final common pathway for the neural control of reproduction. Fibers from GnRH neurons project to the external zone of the median eminence (ME) and release their neuropeptide into the portal blood system; from there, it travels to the pituitary gland to stimulate the synthesis and release of luteinizing hormone, and follicle-stimulating hormone. Under most endocrine conditions, GnRH secretion occurs episodically. Although the pulsatile pattern is essential for normal reproductive function, the mechanism responsible for synchronizing GnRH cell activity remains largely unknown. It is accepted that the timing and degree of GnRH release is controlled by multiple stimulatory and inhibitory factors.

Two RF-amide peptides, kisspeptin and RFamide-related peptide-3 (RFRP-3, also referred to as gonadotropin-inhibitory hormone [GnIH]), are expressed in the hypothalamus and have been shown to stimulate and inhibit GnRH neuronal activity, respectively (Kriegsfeld et al., 2010; Steiner, 2013). Kisspeptins are the peptide product of the *KISS1* gene, stimulate GnRH secretion (Messager et al., 2005), and are critical for reproductive function (De Roux et al., 2003). No other factor has been shown to be as potent a stimulator of GnRH neurons as kisspeptin (Steiner, 2013). In addition, the kisspeptin neurons in the arcuate nucleus (ARC) co-express the endogenous opioid dynorphin, which is inhibitory to pulsatile GnRH release and neurokinin B (NKB), a stimulator of GnRH release (Foradori et al., 2006; Goodman et al., 2013; Goodman et al., 2007; Wakabayashi et al., 2010). The neurons co-expressing kisspeptin, NKB, and dynorphin have subsequently been coined KNDy (kisspeptin/ neurokinin B/ dynorphin) neurons (Lehman, Coolen & Goodman, 2010) and are found in sheep (Goodman et al., 2007), rats (Burke et al., 2006; True et al., 2011), mice (Navarro et al., 2009), goats (Wakabayashi et al., 2010), and women (Rance, 2009).

There is strong evidence that the KNDy cell group is critical for episodic GnRH release . (Goodman, Coolen & Lehman, 2014). Kisspeptin is the primary output to GnRH neurons, with direct synapses to GnRH soma and fibers in the ME, which express the kisspeptin receptor GPR54 (Clarkson & Herbison, 2006; Hrabovszky et al., 2010; Magee et al., 2009; Messager et al., 2005; Ramaswamy et al., 2008). The neuropeptide NKB acts within the KNDy network to initiate GnRH pulses and dynorphin acts to inhibit KNDy neural activity, thus terminate each pulse (Goodman et al., 2013). The proposed actions of NKB are supported by reports that the stimulatory actions of the NK3R agonist senktide on GnRH secretion are mediated by kisspeptin release from KNDy neurons in several species (Goodman, Coolen & Lehman, 2014; Navarro et al., 2011; Ramaswamy, Seminara & Plant, 2011) and that intracerebroventricular administration of NKB stimulates cell activity in the ARC of the goat and increases LH pulse frequency in the ewe (Goodman, Coolen & Lehman, 2014; Wakabayashi et al., 2010). Acting as the brake of the KNDy network is the endogenous opioid dynorphin. The general opioid receptor antagonist naloxone prolongs each GnRH pulse in ovariectomized ewes. (Goodman et al., 1995), and central administration of the *κ*-opioid receptor antagonist nor-binaltorphimine increases the frequency of multiple-unit activity in the ARC of ovariectomized goats (Wakabayashi et al., 2010) and increases LH pulse frequency in the ewe (Goodman et al., 2013).

Working through the G protein-coupled receptor GPR147, RFRP-3 has been implicated in the negative regulation of LH secretion (Ubuka et al., 2013). The RFRP-3 neurons are mainly localized in the dorsomedial hypothalamic nucleus (DMN), from which fibers project to GnRH neurons in the preoptic area (POA) in mammals (Smith et al., 2008a; Ubuka et al., 2012). In rodents, GPR147 mRNA is expressed in a subset of GnRH neurons (Rizwan et al., 2012). The main inhibitory effect of RFRP-3 appears to be at the level of GnRH neurons in rodents, as indicated in hamsters (Kriegsfeld et al., 2010), rats (Johnson, Tsutsui & Fraley, 2007), and mice (Ducret, Anderson & Herbison, 2009). These findings, along with GnRH cell recordings, strongly suggest a direct action of RFRP-3 on GnRH neurons (Glanowska, Burger & Moenter, 2014). Studies *in-vivo* support this data, in which intravenous administration of RFRP-3 decreased plasma LH concentrations during the late follicular phase of the estrous cycle in intact ewes and during an estradiol benzoate-induced LH surge in ovariectomized ewes (Clarke et al., 2008).

The KNDy network and RFRP-3 neurons have also been implicated in steroid hormone feedback onto GnRH neurons. In females, tonic negative feedback effects of estrogen and progesterone prevail throughout most of the ovarian cycle. In the late follicular phase of the cycle, a neuroendocrine switch occurs, and a transient, estrogen-induced positive feedback effect causes the preovulatory surge in GnRH/LH (Pau et al., 1993). The surge in LH secretion causes ovulation. Because GnRH neurons do not possess the requisite sex steroid receptors (Herbison & Theodosis, 1992; Skinner, Caraty & Allingham, 2001), feedback signals to these neurons rely on transmission through other steroid-receptive cells within the brain. The majority of KNDy neurons express estrogen receptor alpha and progesterone receptors (Franceschini et al., 2006; Lehman, Coolen & Goodman, 2010; Smith et al., 2007). The level of expression of kisspeptin, dynorphin, and NKB neuropeptides are responsive to gonadal steroid levels (Foradori et al., 2002; Foradori et al., 2005; Ruiz-Pino et al., 2012; Smith et al., 2009). Similarly, the RFRP-3 expressing cells also express gonadal hormone receptors, respond to estrogen levels, and have GnRH-contacting projections (Gibson et al., 2008; Molnar et al., 2011; Poling et al., 2012).

Although there is evidence that the KNDy network and RFRP-3 are conserved across species, to date, there has been no investigation of these cell groups in the economically relevant adult bovine. Thus the primary goal of this work was to determine the distribution of kisspeptin, dynorphin, and RFRP-3 in the hypothalami of bovine during either the periestrus or diestrus stage of the estrous cycle. In addition, we examined the connectivity of kisspeptin and RFRP-3 fibers onto GnRH soma in both periestrus and diestrus animals.

## Material and Methods

### Ethics Statement

The University of Tennessee Animal Care and Use Committee approved all animal procedures (Protocol 1982-0111).

### Animals

Six adult (2–4 years old), second parity non-lactating Holstein bovine were maintained in an open free stall barn with free access to water and fed a total mixed ration once daily. To synchronize the estrous cycles, bovine at random stages of their estrous cycle were given an initial dose of GnRH (Cystorelin, Merial; 2 ml; 50 µg/ml; IM) and fitted with a progesterone-releasing intravaginal device (Eazi-Breed CIDR, Zoetis; 1.38 g progesterone) that was left in place for 7 days. On day 7, the intravaginal device was removed and bovine were given synthetic prostaglandin cloprostenol (Estrumate, Merck Animal Health; 2 ml; 250 µg/ml; IM) to induce luteolysis. Three days after the first dose of cloprostenol, all bovine were given a second dose of GnRH (Cystorelin; 2 ml; 50 µg/ml; IM). Six days after the second dose of GnRH, uteri and ovaries were examined by transrectal palpation and ultrasonography (MyLab Five VET, Esaote; 5 mHz linear rectal probe) to confirm the presence of a corpus luteum and an antral follicle. All six animals were randomly assigned to one of two groups: a diestrus group (DE, *n* = 3) and a periestrus group (PE, *n* = 3). Twenty four hours after transrectal ultrasound examination, those selected as part of the PE group were given a second dose of cloprostenol (Estrumate; 2 ml; 250 µg/ml; IM) to elicit luteolysis (DesCoteaux et al., 2010). In the bovine, periestrus is a period of the estrus cycle encompassing −3 days to +4 days from estrus (DesCoteaux et al., 2010). Estrus behavior and LH hormone levels were not determined; therefore, this period was defined as a non-progesterone dominated phase of the bovine estrous cycle.

Euthanasia and sample collection were carried out 24 h after transrectal examination for animals in the DE group and 24 h after the second dose of cloprostenol for animals in the PE group. Before termination, blood samples were taken by jugular venipuncture, and harvested plasma was stored at −20 °C until assayed using the estradiol (ImmunChem Double antibody, MP Biomedicals) and progesterone Coat-A-Count Kit (Siemens Medical Solutions Diagnostics). Both assays have been previously validated in the bovine (Karcher, Beitz & Stabel, 2008; Tanco et al., 2012). Bovine were euthanized and hypothalami collected and fixed as previously described (Magee et al., 2009). Briefly, animals were given 50,000 IU heparin (Sigma) i.v. and euthanized with an i.v. dose of sodium pentobarbital (20 mg/kg) 15 min later. After decapitation, the carotid arteries were catheterized, basilar arteries clamped off, and heads perfused with 6 liters of 4% paraformaldehyde in 0.1 M phosphate-buffered saline, with 0.1% sodium nitrite, pH 7.4. Hypothalamic blocks were dissected with the following margins: rostrally–rostral border of the optic chiasm; caudally–rostral to the mammillary bodies; laterally—1 cm off midline, lateral to the optic chiasm; and dorsally—0.5 cm above the third ventricle. Tissue was stored in 4% paraformaldehyde at 4 °C overnight and then placed in 30% sucrose at 4 °C until infiltration was complete. Thick (50 µm) frozen coronal sections were cut in series of six and stored at −20 °C in a cryopreservative solution until being processed immunohistochemically.

### Immunohistochemistry

Neuropeptides were detected using a modified avidin-biotin-immunoperoxidase protocol with 3,3′-diaminobenzidine as chromogen (brown reaction product). The immunohistochemistry procedure was carried out on free-floating sections at room temperature, except for incubation with primary antibodies, which were performed at 4 °C, as previously described (Foradori et al., 2006). Briefly, sections were repeatedly washed in 0.1 M phosphate buffer with 0.9% saline (PBS) to remove cryoprotectant. Sections used for the detection of kisspeptin were subjected to high-temperature antigen retrieval as previously described (Skinner, Caraty & Allingham, 2001). After washing, the sections were placed in a 1% hydrogen peroxide (Sigma) solution for 10 min to remove endogenous peroxidase activity for chromogen detection. The sections were then washed and incubated for 1 h in PBS containing 4% normal donkey serum (Jackson Laboratories) and 0.4% Triton X-100 (Sigma; PBSTX). Alternate sections were then incubated with polyclonal antibodies against GnRH (rabbit host; 1:10,000; PA1-121; ThermoFisher Scientific), kisspeptin (rabbit host; 1:30,000; generously provided by Alain Caraty, Institut National de la Recherche Agronomique), RFRP-1/3 (rabbit host; clone GA197; 1:30,000; generously provided by Greg Anderson, University of Otago School of Medical Sciences), or DYN A 1–17 (rabbit host; 1:20,000; IHC 8730; Peninsula Laboratories) for 48 h in PBTX. Following incubation, chromogen sections were washed and then placed in a solution of PBSTX with biotinylated donkey anti-rabbit IgG (1:1,000; Jackson Laboratories) for 1 h. The sections were washed and incubated for 1 h in avidin-biotin-HRP complex (1:1,000; Vector Laboratories). Neuropeptides were visualized using 3,3′-diaminobenzidine and 0.003% hydrogen peroxide as substrate. Control sections for the immunohistochemistry procedure included omission of each of the primary antibodies from the immunostaining protocol, which resulted in a complete absence of staining for the corresponding antigen. In addition, pre-absorption controls were performed for each of the antibodies. In each case, pre-incubation of the diluted antiserum with nanomolar concentrations of purified antigen (Phoenix Pharmaceuticals) was shown to be sufficient to eliminate all specific staining in bovine hypothalamic sections. It should be noted that the rabbit anti-RFRP1/3 clone was generated from the precursor peptide, which produces both RFRP-1 and RFRP-3 (Yano et al., 2003); therefore, labeling the precursor effectively defines RFRP-1/RFRP-3-expressing neurons (Rizwan et al., 2009). The dynorphin A 1–17 antibody shows cross-reactivity with dynorphin A 1–13 and none with other prodynorphin derivatives such as dynorphin A 1–8, *α*-neo-endorphin, leu-enkephalin, and dynorphin B (Foradori et al., 2002). The kisspeptin antibody was made from the peptide YNWNSFGLRY-NH2 (kp10), corresponding to amino acid residues 43–52 of mouse metastin. This sequence has high homology to the predicted bovine protein (GenBank accession number AB466319.1). RFRP precursor peptide antiserum was generated to amino acids 119-132 of the rat RFRP precursor peptide sequence (PSLPQRFGRTTARR), which matches that of the bovine (Rizwan et al., 2009; Tsutsui & Ubuka, 2013).

### Dual-labeled immunofluorescence

To investigate the possible interaction between GnRH-expressing cells and cells immunoreactive for RFRP-1/3 or kisspeptin, dual-immunofluorescence was performed. As stated above, sections were washed and incubated with the monoclonal antibody mouse anti-GnRH (1:3,000; SMI 41, Biolegend) and either rabbit antibody RFRP-1/RFRP-3 (1:10,000) or kisspeptin (1:10,000) for 48 h in PBTX at 4 °C. After primary incubations, sections were washed and incubated in Alexa Fluor 488 or Cy3 conjugated to donkey anti-rabbit or mouse IgG, respective to primary host. Sections processed to detect GnRH and kisspeptin were put through antigen retrieval as described above. Omission of one or both of the primary antibodies completely removed all corresponding staining.

### Tissue analysis

The distribution of immunoreactive cells and fibers was examined in a series of every sixth section (50 µm thick each) through the preoptic area and hypothalamus of each animal. Images of labeled material were captured using a digital camera (QImaging Retiga 2000R) attached to a Nikon microscope (Eclipse E800M), and NIS-Elements software version 4.11 (Nikon). Images were imported into Adobe Photoshop CS6 (Adobe Systems) and were not altered in any way except for minor adjustments of brightness and contrast. The number of cells identified by immunohistochemistry for a nucleus/region was estimated by summing the total number of cells observed within the borders of each nuclei/region in three representative sections from each animal. Sections were atlas matched (Swanson, 1992; Welker, JI & Noe, 2016), and areas were identified by examining cresyl violet-stained tissue of alternate serial cut sections, under bright-field microscopy.

GnRH fiber densities and intensity were evaluated by two ways. Fluorescent intensity was measured by arbitrary density units (ADU) above an established threshold. Fiber density was determined by examining the number of pixels in a defined area (250-µm^2^ square) to determine the degree of ME taken up by immunofluorescent fibers positive for GnRH. The fiber density value consisted of the area (in pixels) covered by labeled fibers divided by total area (in pixels) within the boundaries (Nikon Elements). The percentage of GnRH immunoreactive (-ir) cells with close apposition with kisspeptin or RFRP 1/3-ir processes were calculated. Each identified GnRH-ir soma was examined under a Nikon A1 confocal with an Eclipse TE2000-E microscope under a 40× objective. Digital images were acquired using the NIS Elements AR software. If there were no discernable pixels between GnRH-positive cell bodies and RFRP-3 or kisspeptin fibers, the cell was considered to be in close apposition to the fiber. All image acquisition and analyses were performed blind to hormone status of animal.

### Statistical analysis

Results are reported as mean ± SEM. Data was analyzed using the unpaired Student *t* test, and non-normally distributed data points were analyzed using the Mann–Whitney-Wilcoxon test (SAS software 9.3; SAS Institute Inc.). Significance was assumed when the probability of values differing by chance alone was 0.05 or less.

## Results

### Hormone levels and ovarian structures

**Figure 1:**
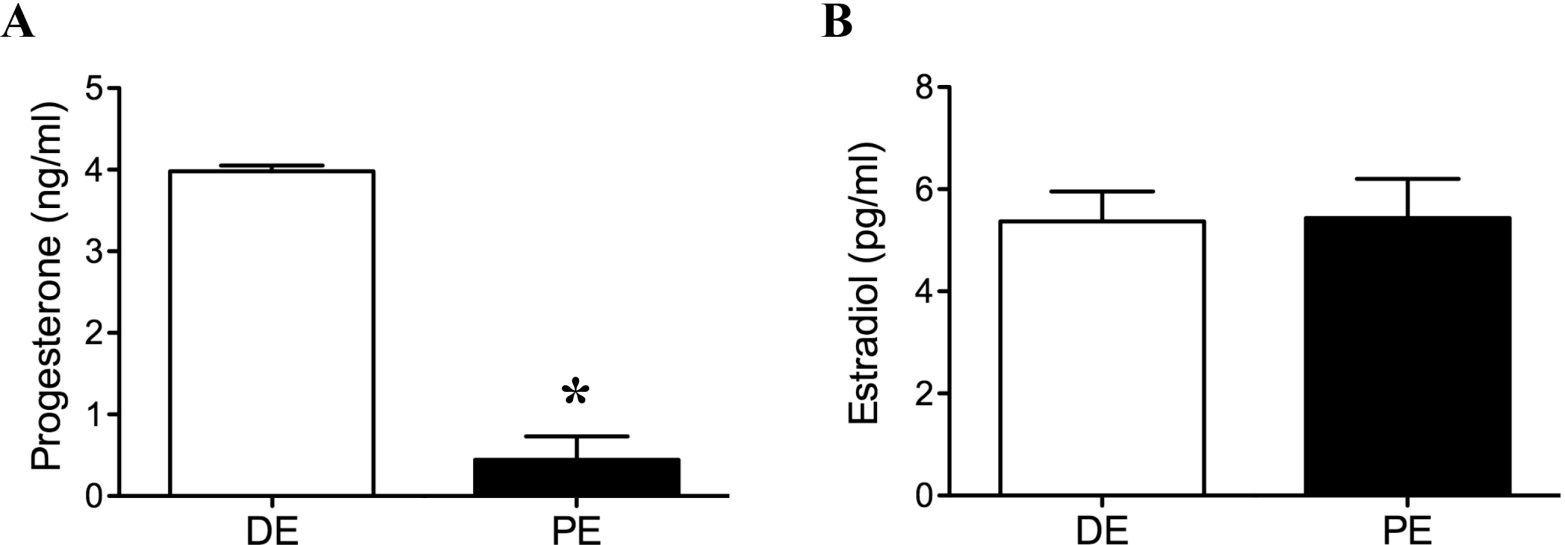
Plasma progesterone (A) and estradiol (B) concentrations at the time of tissue collection in diestrus (DE; luteal phase; *n* = 3) and periestrus (PE; non-luteal phase; *n* = 3) animals. Plasma progesterone concentration was higher in DE animals when compared to PE animals (*P* = 0.03; ^∗^ = statiscally significant). There was no difference in estradiol concentration between groups.

Progesterone concentration at the time of euthanasia in the DE group was higher (luteal phase) than in the PE group (non-luteal phase; *P* = 0.03, Fig. 1). All DE animals had progesterone levels below 1 ng/ml. There was no difference in estradiol concentration at the time of euthanasia between animals in the DE and PE groups (*P* = 0.94, Fig. 1). All animals were found to have a corpus luteum and a follicle >10 mm in diameter at the time of transrectal ultrasound examination performed six days after the second dose of GnRH (24 h before cloprostenol administration).

### Distribution of GnRH immunoreactivity in the PE and DE bovine

**Figure 2:**
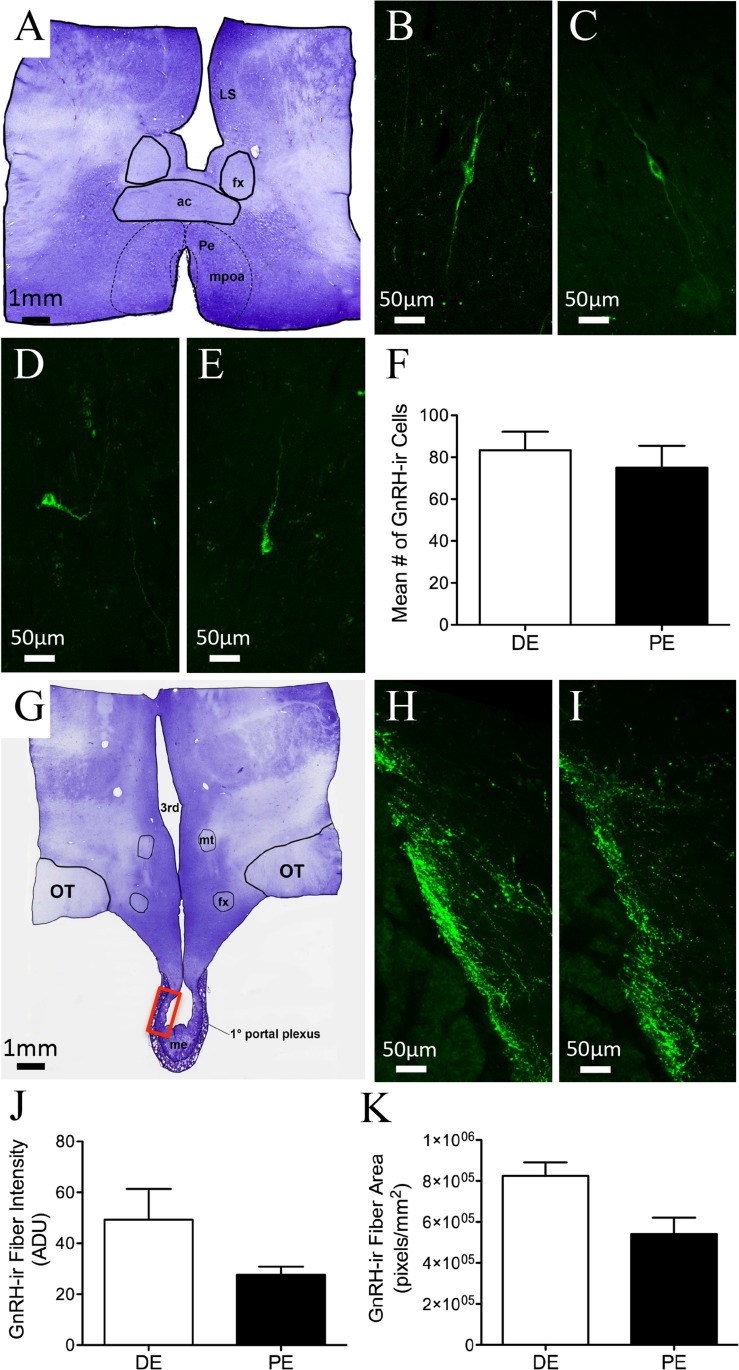
GnRH-ir in the bovine POA and ME. (A, G) Low-power images of a representative crystal violet stained sections, red box depicting ME area of photomicrographs. (B–E) POA bipolar and unipolar GnRH-ir cells in DE (B, D) and PE (C, E) animals. (F) Histogram depicting mean number (±SEM) of GnRH-ir cells identified in the POA of DE and PE animals. (H–I) ME representative images of GnRH-ir fibers in DE (H) and PE (I) animals. (J–K) Histograms depicting mean (±SEM) arbitrary density units (J) and total area (K) of GnRH-ir fibers in the ME of DE and PE animals.

GnRH-ir soma were distributed throughout the bovine POA and mediobasal hypothalamus (MBH). Rostrally, GnRH-ir cells were identified in the diagonal band of Broca (dbB) and the medial POA (mPOA; Figs. 2A–2E; Table 1). They were also found concentrated along the midline in areas of the organum vasculosum of the lamina terminalis (OVLT), with a few cells identified in the medial septum. Perikarya were also found, to a lesser degree, in the ventral anterior hypothalamic area (AHA) and MBH. The number of GnRH neurons varied among regions; however, the majority of GnRH neurons were found in the POA. GnRH-ir fibers were identified throughout the bovine hypothalamus, with a high density of fibers found along the dbB, in and around the mPOA, lateral hypothalamus, OVLT, surrounding the bed nucleus of the stria terminalis (BNST), AHA, and lateral septum. Fibers were also present in the ventrolateral AHA and along the ventral-lateral borders of the third ventricle. The largest density of GnRH-ir fibers was identified in the MBH directed toward the external zone of the ME and into the infundibular stalk and pars tuberalis (Figs. 2G–2I). GnRH-ir cells and fibers were analyzed in the hypothalamus and ME of animals in DE and PE. No difference was found in the number of GnRH-ir cells (Fig. 2F, *P* = 0.57) in the POA. Nor was there a measurable difference in the degree of ADUs above threshold (Fig. 2J and *P* = 0.15) and area of GnRH-ir fibers in the ME (Fig. 2K, *P* = 0.051) between animals in the DE and PE groups. GnRH-ir cell number and fiber area and density had similar distribution and level regardless of progesterone concentration in bovine.

**Table 1:** Neuropeptides soma and fibers in the hypothalamus of DE and PE bovine.

	Neuropeptides							
	GnRH		Dynorphin		Kisspeptin		RFRP-1/3	
	Soma	Fibers	Soma	Fibers	Soma	Fibers	Soma	Fibers
Brain Areas	DE/PE	DE/PE	DE/PE	DE/PE	DE/PE	DE/PE	DE/PE	DE/PE
dbB	+ + ∕ + +	+ + ∕ + +	−∕ −	−∕ −	−∕ −	+∕ +	−∕ −	+∕ +
Medial Septum	+∕ +	+∕ +	−∕ −	−∕ −	−∕ −	−∕ −	−∕ −	+∕ +
Lateral Septum	−∕ −	+∕ +	−∕ −	−∕ −	+∕ +	+∕ +	−∕ −	+∕ +
mPOA	+ + + ∕ + + +	+ + ∕ + +	+∕ +	+∕ +	+∕ +	+ + ∕ + +	−∕ −	+∕ +
OVLT	+ + + ∕ + + +	+ + + ∕ + + +	+∕ +	+∕ +	−∕ −	+∕ +	−∕ −	+∕ +
AHA	+∕ +	+ + ∕ + +	+ + ∕ + +	+ + ∕ + +	−∕ −	+∕ +	−∕ −	+∕ +
BNST	−∕ −	+∕ +	+∕ +	+∕ +	−∕ −	+∕ +	−∕ −	−∕ −
PVN	−∕ −	−∕ −	+ + + ∕ + + +	+ + ∕ + +	−∕ −	+∕ +	+∕ +	+∕ +
SON	−∕ −	−∕ −	+ + + ∕ + + +	+ + ∕ + +	−∕ −	−∕ −	−∕ −	+∕ +
Basolateral MBH	+∕ +	+ + ∕ + +	−∕ −	+∕ +	−∕ −	−∕ −	−∕ −	+∕ +
DMN	−∕ −	+∕ +	+∕ +	+∕ +	−∕ −	+∕ +	+ + ∕ + +	+∕ +
VMN	−∕ −	+∕ +	−∕ −	+∕ +	+∕ +	+∕ +	−∕ −	+∕ +
PrVN	−∕ −	+∕ +	+∕ +	+ + ∕ + +	+∕ +	+∕ +	+∕ +	+∕ +
ARC	−∕ −	+∕ +	+ + ∕ +	+ + ∕ +	+∕ + + +	+ + ∕ + + +	−∕ −	+∕ +
ME	−∕ −	+ + ∕ + +	−∕ −	+∕ +	−∕ −	+∕ +	−∕ −	−∕ −

**Figure 3:**
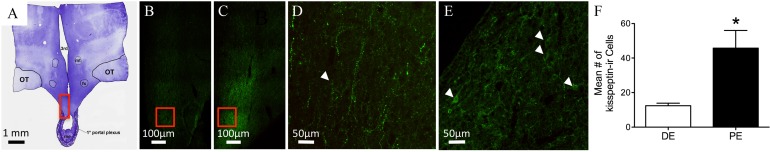
Kisspeptin in the bovine arcuate nucleus. (A) Low-power image of a representative crystal violet stained section with red box depicting area of the arcuate nucleus of photomicrographs. (B–E) Representative images of kisspeptin-ir cells in DE (B, D) and PE (C, E) animals. Red box in B and C depict areas of images D and E, respectively. (F) Histogram depicting mean number (±SEM) of kisspeptin-ir cells identified in DE and PE bovine. (^∗^ = statistically significant, *p* = 0.04). White arrowheads indicate representative somas.

### Distribution of kisspeptin immunoreactivity (-ir) in the PE and DE bovine

Kisspeptin-ir was examined in the hypothalamus of six bovines (Figs. 3A–3C; Table 1). Examination of serial sections revealed large clusters of kisspeptin-ir soma in the ARC. A few scattered kisspeptin-ir cells were also localized to the preoptic periventricular zone of the hypothalamus adjacent to the third ventricle. However, the exiguous number of kisspeptin-ir cells was present in only two of the animals (one in the DE group and one in the PE group); therefore, analysis of these cell populations was not warranted. Kisspeptin-ir cells appeared to be at the highest density in the ARC. Immunoreactive cells were distributed throughout the rostrocaudal extent of the ARC. A dense network of kisspeptin-ir varicose fibers surrounded kisspeptin-ir soma in the ARC. Cells reached from the ARC into the ventromedial nucleus (VMN; Figs. 3A–3C). Immunoreactive fibers were also identified in the dbB, OVLT, lateral hypothalamus, lateral septum, paraventricular nucleus of the hypothalamus (PVN), and surrounding the bed nucleus of the BNST. Kisspeptin-ir cell number was analyzed in the ARC of DE and PE cattle. The number of kisspeptin-ir cells was higher in the PE group compared to the DE group (*P* = 0.04, Fig. 3F).

### Distribution of dynorphin immunoreactivity (-ir) in the PE and DE bovine

**Figure 4:**
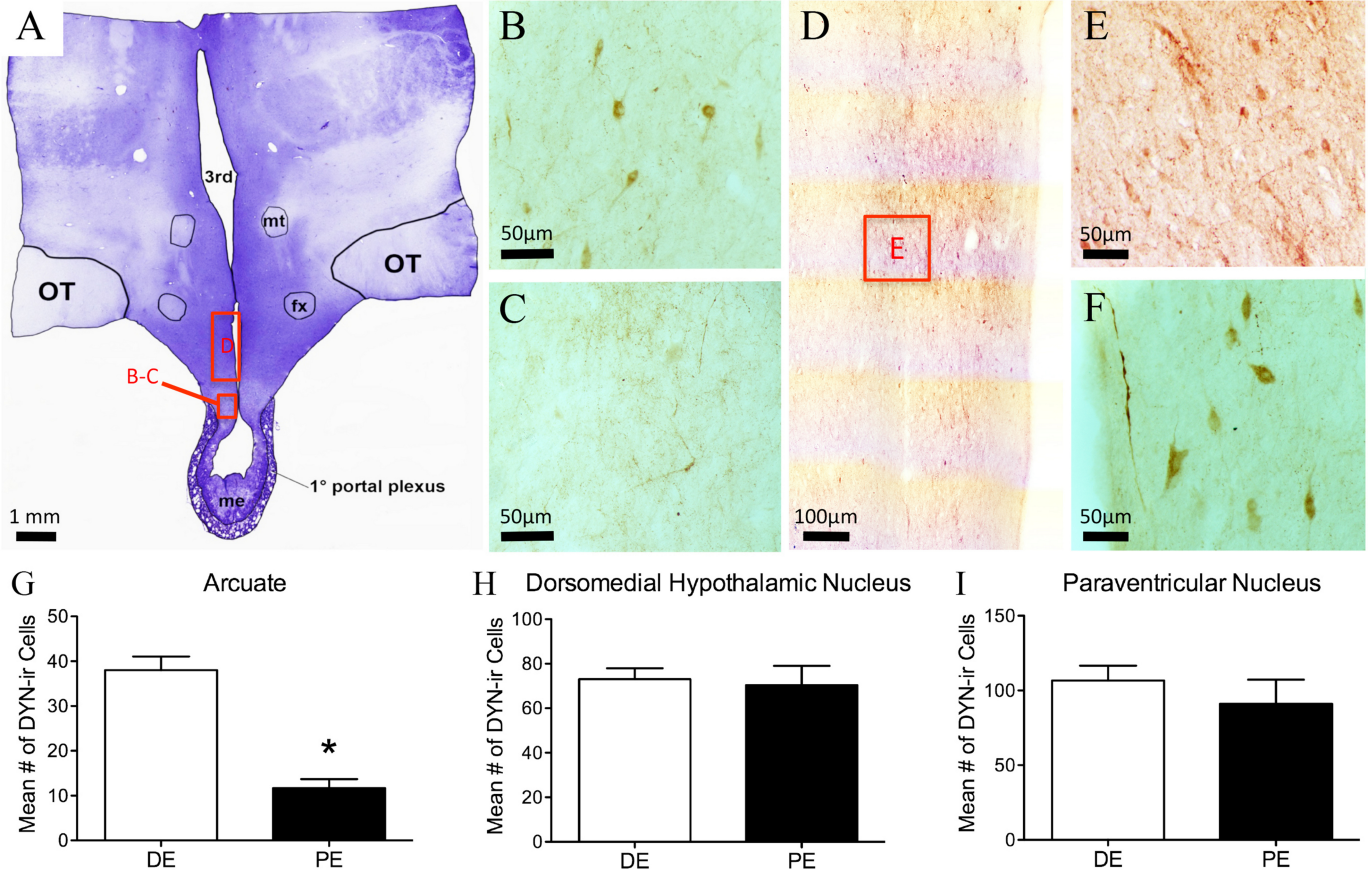
Dynorphin (DYN) in the bovine hypothalamus. (A) Low-power image of a representative crystal violet stained section with red boxes depicting areas of photomicrographs. (B–C) Representative images of DYN-ir cells in the arcuate nucleus of the diestrus (B) and periestrus (C) animals. Representative low- (D) and high-power (E) images of DYN-ir cells in the dorsomedial nucleus and (F) paraventricular nucleus of a DE animal. Histogram depicting the mean number (±SEM) of DYN-ir cells identified in the diestrus and proestrus bovine (G) arcuate nucleus, (H) dorsomedial nucleus and (I) paraventricular nucleus. (^∗^ = statistically significant, *p* = 0.001).

Dynorphin-ir was seen in two morphologically distinct types of cells: magnocellular neurons (mean somal diameter = 23.6 ± 3.8 µm) seen in the PVN and supraoptic nucleus (SON); and parvicellular neurons (mean somal diameter = 11.2 ± 2.7 µm) seen in the BNST, lateral hypothalamus, dorsomedial nucleus of the hypothalamus (DMH), and ARC (Fig. 4 and Table 1). Dynorphin-ir in the BNST was found in a limited population directly lateral and dorsal to the anterior commissure. Dynorphin-ir fiber labeling was also seen in the BNST, predominantly in the portion of this nucleus directly medial to the anterior commissure.

Dynorphin-ir cells were located throughout the rostral-caudal extent of the ARC, with most of the cells localized to the dorsal regions (Figs. 4B–4C). The number of dynorphin-ir cells in the ARC nucleus was higher in the DE group compared to the PE group (38.0 ± 0.1 and 11.6 ± 7, respectively, *P* = 0.001; Fig. 4G). There was no difference between the DE and PE groups in the number of dynorphin-ir cells in the DMH (73 ± 4.9 vs. 70.3 ± 8.7; *P* = 0.80) or the PVN (106.7 ± 10.0 vs. 91.0 ± 16.2; *P* = 0.45; Figs. 4H–4I). Higher numbers of dynorphin-ir cells in the ARC nucleus were associated with high progesterone levels in cattle.

### Distribution of RFRP1/3 immunoreactivity (-ir) in the PE and DE bovine

**Figure 5:**
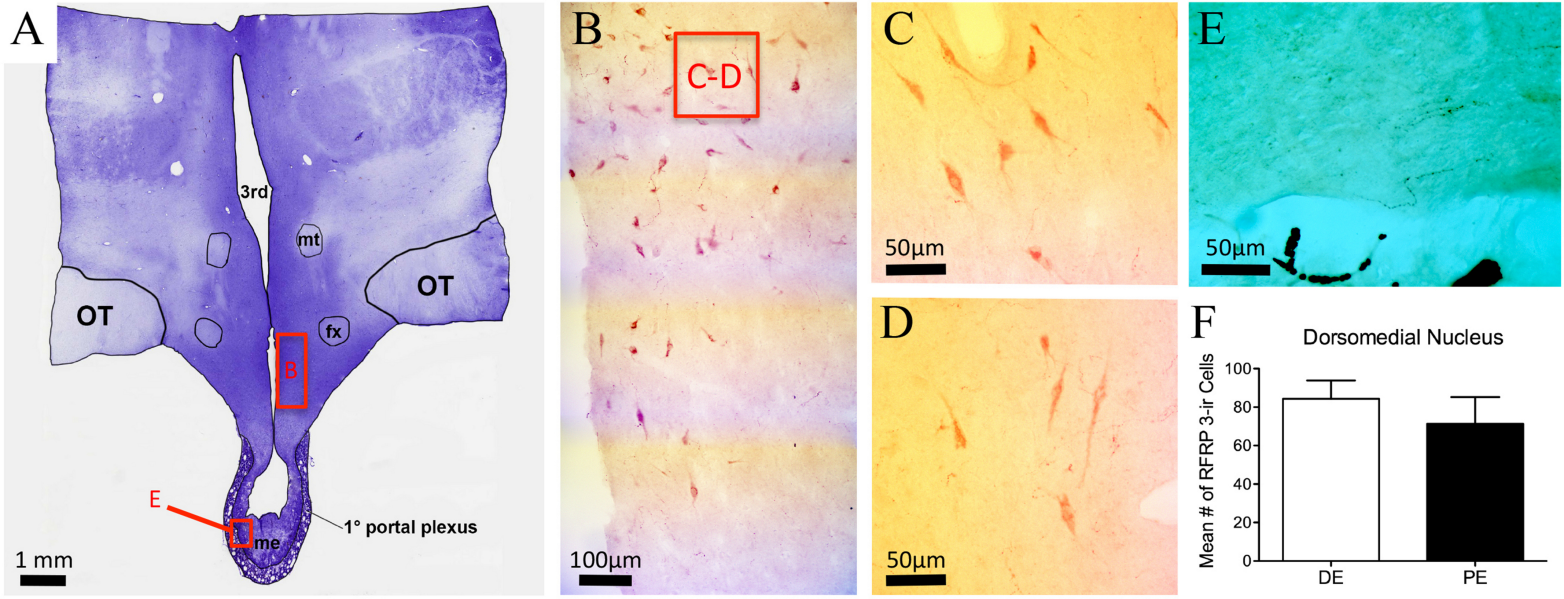
RFRP-3 in the bovine dorsomedial nucleus of the hypothalamus. (A) Low-power image of a representative crystal violet stained section with red box depicting area of photomicrographs. (B–D) Representative low- (B) and high-power (C–D) images images of RFRP-3-ir cells in the dorsomedial nucleus of the hypothalamus of the diestrus (C) and periestrus (D) animals. (E) Image of RFRP-3-ir fibers in ME. (F) Histogram depicting mean number (±SEM) of RFRP-3-ir cells identified in the DE and PE bovine dorsomedial nucleus of the hypothalamus.

RFRP-1/3-ir cell bodies were observed only within the DMH and distributed dorsally into the ventral and lateral borders of the PVN (Figs. 5A–5D; Table 1). Cells were also distributed medially into the periventricular nucleus (PrVN). The cells were scattered throughout this region and exhibited a neuronal, multipolar morphology. The distribution and number of RFRP-1/3-ir neurons were not different between DE and PE female animals (84.3 ± 9.5 vs. 71.3 ± 13.9, respectively; Fig. 5E; *P* = 0.48). Scattered fibers were detected in the horizontal and vertical limbs of the dbB, lateral septum, POA (including the region around the OVLT), PrVN, AHA, and rostral aspects of the lateral hypothalamus. In the medial hypothalamus, fibers were observed only in the DMH and ventromedial hypothalamus. Only a very few fibers were seen in the ARC and ME.

### GnRH connectivity with kisspeptin or RFRP1/3 fibers in the PE and DE bovine

**Figure 6:**
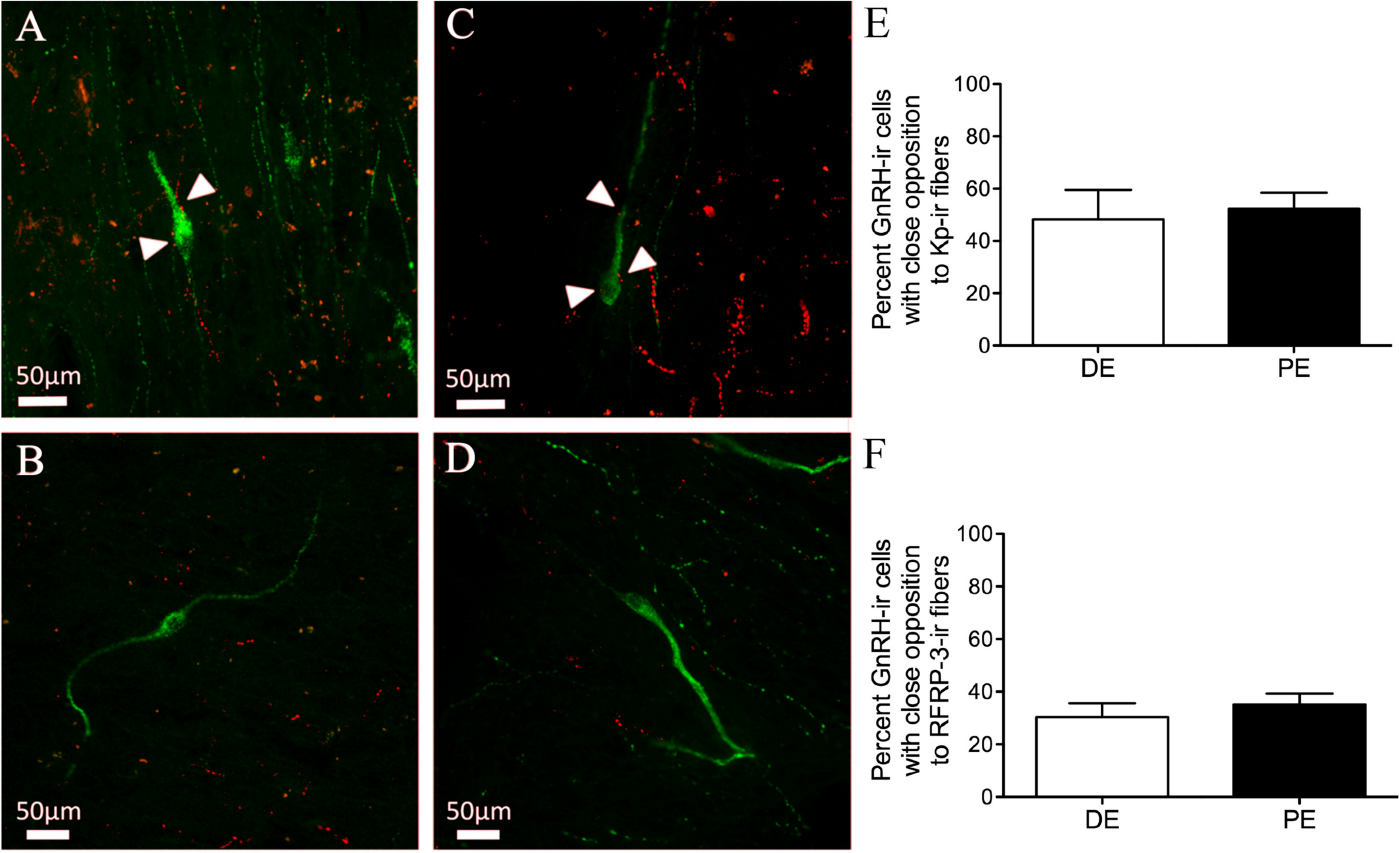
Neuropeptide connectivity to GnRH in the bovine hypothalamus. (A–D) Representative images of GnRH-ir (green) cells with (A, C) or without (B, D) Kisspeptin (A–B) or RFRP-3 (C–D) -ir fibers (red) in close apposition (white arrows). Histograms depicting mean percent (±SEM) of GnRH-ir soma identified to have close apposition with Kisspeptin-ir (E) or RFRP-3-ir (F) in DE and PE animals.

Finally, we sought to determine the levels of connectivity between GnRH and RFRP-3 or kisspeptin cells in the bovine hypothalamus. Kisspeptin-ir fibers and GnRH-ir neuron contacts were identified in the dbB, mPOA, and MBH (Figs. 6A–6B and Table 1). The percent of GnRH-ir neurons with kisspeptin-ir fiber contacts did not vary for each region. There was no identified difference in the percentage of GnRH neurons with kisspeptin contacts between DE and PE animals (48.7 ± 11.3 vs. 52.3 ± 6.2, respectively; Fig. 6E). The total mean percentage of GnRH-ir neurons in close apposition with kisspeptin-ir fibers was 49.8%.

Both RFRP-3-ir fiber and GnRH-ir neuron contacts were identified in the dbB, mPOA, and MBH (Figs. 6C–6D). The percent of GnRH-ir neurons with RFRP-3-ir fiber contacts did not vary by brain region and there was no identifiable difference in the percentage of GnRH neurons with RFRP-3 contacts between DE and PE animals (30.4 ± 5.2 vs. 35.1 ± 4.2, respectively; Fig. 6F). The total mean percentage of GnRH-ir neurons in close apposition with RFRP-3 fibers was 31.3%.

## Discussion

With regard to the changing patterns of GnRH/LH release during the ovarian cycle and due to the excitatory effect of kisspeptin and inhibition by RFRP-3 and dynorphin, we hypothesized that changes in the immunoreactivity of neuropeptides of the KNDy cells of the ARC and RFRP-3, primarily in the DMN, would correlate to hormone changes related to the ovarian cycle in the bovine.

Bovine GnRH-ir somal and fiber distribution were similar to previous reports in the bovine, with a majority of GnRH neurons found in the mPOA adjacent to the OVLT (Leshin et al., 1988). These cells formed a continuum rostrally with immunoreactive neurons in the dbB and medial septum, and caudally with cells in the ventrolateral anterior hypothalamus and lateral hypothalamus. Relatively, few cells were seen in the anterior hypothalamic area and medial basal hypothalamus. There was no discernable difference in the mean number of GnRH-ir cells between the PE and DE groups. A previous report on GnRH mRNA expression in the heifer stated that mid-luteal animals displayed a reduction in the number of GnRH mRNA-expressing cells and number of grains per cell when compared to ovariectomized animals (Weesner et al., 1993). These findings were most likely due to the extreme differences in gonadal hormones when comparing intact and ovariectomized animals, which was not endemic of the current study.

Similar to previous reports, bovine GnRH fibers were mainly found in two major pathways: a ventrolateral projection above the optic tract in the anterior and lateral hypothalamus and a less prominent periventricular pathway along the third ventricle (Leshin et al., 1988). Differences in GnRH-ir fiber density in the ME were not identified in PE and DE animals. These findings are not surprising since the only previous reports of differences were found in narrow windows comparing animals just before and after the GnRH surge; other groups have reported increased ME GnRH-ir during proestrus (Kaur, Heera & Srivastava, 2002; Prevot et al., 1998) and others a decrease (Polkowska & Jutisz, 1979; Rubin & King, 1995). In the monkey, maximum concentration of GnRH fibers were identified during the early and middle follicular phases, with a decrease seen during the late follicular and ovulatory phases and an increase during the luteal and early follicular phases (Barry & Croix, 1978). In the ME of the sheep, there has been a report of decreased GnRH-ir 24 h after ovulation (Polkowska, Dubois & Domanski, 1980). Samples were not taken during this time in the current study to observe the predicted drop after the preovulatory surge. In addition, the limited animal number in each group, while large enough to identify significant changes in several metrics (hormone and neuropeptide immunoreactivity), may have resulted in a statistical power insufficient to identify possible smaller changes.

The distribution of RFRP-3 in the bovine hypothalamus, overall, matched well with descriptions of RFRP-3 distribution in the mouse (Ukena & Tsutsui, 2001), goat (Jafarzadeh Shirazi et al., 2014), sheep (Clarke et al., 2008), rat (Rizwan et al., 2009; Yano et al., 2003), sparrow (Calisi, Rizzo & Bentley, 2008), hamster (Ubuka et al., 2012), o’possum (Harbid et al., 2013), and primate (Smith & Clarke, 2010). Bovine RFRP-3 cells were located mainly in the DMN, but also distributed into the PVN and PrVN. The number of RFRP-3-ir cells was not different between PE and DE animals. The lack of change in RFRP-3-ir in the bovine was not surprising considering previous studies have reported conflicting findings. Expression of RFRP-3 mRNA in mice DMN is inhibited by E2 (Molnar et al., 2011) but was not found to be different in diestrus, ovariectomized, or ovariectomized plus E2 rats (Quennell et al., 2010). More recently, Salehi et al. (2013) reported the expression of RFRP-3 mRNA was elevated in diestrus rats when compared to proestrus animals. In contrast, female non-human primates display lower neuropeptide VF precursor mRNA (the gene for RFRP in the primate) expression during the luteal phase than in the follicular phase of the menstrual cycle, and in ewes, the expression is reduced during the preovulatory period. (Smith et al., 2010). A recent report in the goat identified a greater number RFRP-ir cells in the DMN during the luteal stage compared to the follicular stage (Jafarzadeh Shirazi et al., 2014). The most profound and reproduced changes in RFRP-3 protein levels in the DMN have been in comparison of breeding and nonbreeding animals (Harbid et al., 2013; Smith et al., 2008a). Perhaps somewhat unique to previous reports, the current findings compared animals with equivalent estrogen levels and dissimilar progesterone levels. While not equivalent, in studies where RFRP-3 expression was measured in pregnant animals, in which progesterone levels were elevated, and nonpregnant animals the relative expression of RFRP-3 mRNA in DMN did not change (Sabet Sarvestani et al., 2014); suggesting dramatic changes in progesterone, alone, do not alter RFRP expression.

Despite the lack of differences in protein regulation, gonadal steroids may alter RFRP-3 activity. In some rodent species and in sheep, a subset of RFRP-3 neurons express estrogen receptors (ERs), but the expression pattern in each species is different. In mice, a very small proportion (19%) of RFRP-3 neurons express ER*α* (Molnar et al., 2011), whereas 40% of RFRP-3 neurons contain ER*α* in female hamsters (Kriegsfeld et al., 2006). During proestrus in hamsters, c-Fos-positive RFRP-3 neurons are reduced and a subcutaneous injection of E2 increased c-Fos labeling in RFRP-3 neurons (Gibson et al., 2008). Although RFRP-3 has been shown to have an effect on LH secretion in male calves (Kadokawa et al., 2009), it is unclear whether endogenous RFRP-3 plays a role in the down regulation of GnRH or LH secretion in the cycling bovine. Gonadal regulation of RFRP-3 neurons may be species- and reproductive stage-dependent. Future work will focus on determining if bovine RFRP-3 neurons also express gonadal steroid receptors and if their activation is responsive to changes steroid levels.

RFRP-3 fibers were distributed throughout the POA and MBH. Fibers were identified in the PrVN and ARC, which match areas with c-Fos expression after central infusion of RFRP in the mouse (Yano et al., 2003). Very few fibers were visualized in the external zone of the ME (Fig. 5E). Although RFRP-3 fibers have been identified in the ME of hamsters (Gibson et al., 2008) and sheep (Clarke et al., 2008), rat (Kriegsfeld et al., 2006; Rizwan et al., 2009; Yano et al., 2003), non-human primates (Smith et al., 2010), and o’possums (Harbid et al., 2013), the amount and distribution into the external zone varies greatly between species (Harbid et al., 2013; Smith et al., 2010). In the bovine, few RFRP-3-ir fibers were localized in the ME, including the external zone; thus, we could predict a limited hypophysiotropic role for RFRP-3 in the bovine. However, RFRP-3 has been shown to inhibit LH release in castrated male calves and cultured anterior pituitary cells of cattle (Kadokawa et al., 2009). This suggests the molecular mechanisms are present for RFRP-3 inhibition of GnRH-induced LH release from bovine gonadotrophs. It remains to be determined if such mechanisms are involved in the regulation of LH in cycling females. Similar findings have been reported in the sheep, a species with more RFRP-3-ir fibers in the external zone of the ME than the bovine. Likewise in the sheep, RFRP-3 has been detected in the portal vasculature (Smith et al., 2012); however, the levels of portal RFRP-3 levels do not vary between luteal and follicular ewes, but are elevated in the non-breeding season when reproductive activity is suppressed (Smith et al., 2012). This suggests an active role for RFRP-3 in the seasonal breeding, but perhaps not estrous cyclicity. Additional work is needed to fully characterize the role RFRP-3 plays in bovine reproduction, but the current findings suggest the RFRP-3 immunoreactivity in the bovine ME is similar to the rodent.

The RFRP-3 terminals appear to make close appositions to GnRH neurons in mice, rats, hamsters, poultry, and sheep (Bentley et al., 2003; Johnson, Tsutsui & Fraley, 2007; Kriegsfeld et al., 2010; Kriegsfeld et al., 2006). In addition, approximately 15–30% of GnRH neurons express mRNA for the RFRP-3 receptor GPR147 in mice (Poling et al., 2012; Rizwan et al., 2012). We report for the first time that roughly 30% of GnRH neurons have RFRP-3 appositions in the female bovine, suggesting that a portion of GnRH neurons in this region respond to RFRP-3. There was no difference in the portion of GnRH cells with RFRP-3 contacts between DE and PE animals. However, the degree of connectivity between RFRP-3 terminals and GnRH is altered in sheep during the breeding season compared to the anestrous season (Smith et al., 2008a). Future work will investigate if similar seasonal differences are present in the bovine.

The distribution of RFRP-3 fiber projections in widespread brain areas of the bovine and other species suggests that RFRP-3 may be involved in a range of physiological functions. Central infusion of RFRP-3 elicits increased food intake in birds, rats, and sheep (Johnson, Tsutsui & Fraley, 2007; Tachibana et al., 2005). Centrally or peripherally administered RFRP-3 also inhibits sexual behavior (Johnson, Tsutsui & Fraley, 2007) and induces anxiety-like behavior (Kaewwongse, Takayanagi & Onaka, 2011) in male rats. Although the present study did not identify a difference in RFRP-3 immunoreactivity or GnRH/RFRP-3 connectivity between PE and DE bovines, there is growing evidence that RFRP-3 plays a large role in seasonal anestrus, puberty onset, and HPG inhibition associated with satiety in seasonal breeders (Harbid et al., 2013; Smith et al., 2008a).

Previous studies on the distribution of dynorphin-ir perikarya and fibers have been carried out in the rat (Fallon & Leslie, 1986), hamster (Neal & Newman, 1989), sheep (Foradori, Goodman & Lehman, 2005), non-human primate (Khachaturian et al., 1985), and human (Abe et al., 1988). However, while relative dynorphin protein levels have been reported in the bovine (Malven et al., 1986; Sonders & Weber, 1987), there have been no studies of the distribution of dynorphin-ir in the bovine hypothalamus. The present findings are in close agreement with earlier studies on the distribution of dynorphin-ir cells and fibers in the POA and hypothalamus of other mammals with large magnocellular dynorphin cells in the PVN and SON; parvocellular cells in the ARC and PrVN; and large fibers densely located in the ARC, PrVN, PVN, and in a circular arrangement in the ventral region of the AHA. Of note, there were fewer dynorphin-ir cells in the POA and more intense immunoreactivity in the PrVN than reported in the ewe (Foradori, Goodman & Lehman, 2005). The highest degree of parvocellular immunoreactivity was seen in the ARC, particularly the middle and caudal regions. There were no differences found in the number of dynorphin-ir cells in the SON, PVN, or PrVN between PE and DE animals. In the ARC, there were an increased number of dynorphin-ir cells in the DE animals. Dynorphin-ir cells in the ovine ARC express progesterone receptors (Foradori et al., 2006) and ovariectomy decreases preprodynorphin mRNA expression in this nucleus (Foradori et al., 2005). In this regard, there is strong evidence that dynorphin participates in progesterone-negative feedback in both pregnant rats (Gallo, 1990) and luteal-phase ewes (Lehman, Coolen & Goodman, 2010). Dynorphin inhibits episodic LH secretion in rats, goats, and sheep (Gallo, 1990; Goodman et al., 2004; Wakabayashi et al., 2010). It is not known whether the same is true in the bovine, but our current findings suggest that bovine ARC dynorphin cells are responsive to progesterone levels and could be playing a part in the gonadal negative feedback on the KNDy “pulse generator” and subsequent GnRH release. In addition to dynorphin’s role in reproduction, it is functionally involved in a variety of neuroendocrine systems, including those mediating feeding. (Baile et al., 1987), water homeostasis (Shimizu et al., 1989), lactation (Kim et al., 1997), and the stress response (Young & Lightman, 1992). Therefore, it is important to have a description of the hypothalamic distribution of this neuropeptide.

As recently reported in juvenile and non-luteal bovine kisspeptin-ir cells were primarily found in the ARC, with an elongated distribution from the lateral edges of the ME ventrally to infiltrating the borders of the ventromedial nucleus, dorsally (Alves et al., 2015; Cardoso et al., 2015). As with other species, the majority of cells were found in the middle and caudal regions of the ARC. As stated previously, there were little to no kisspeptin-ir cells in the POA of the bovine. In rodents, KISS1 mRNA-expressing cells are located in the ARC and the POA (Clarkson & Herbison, 2006). In sheep, goats, and deer, the majority of kisspeptin-ir has been reported in the ARC, with a smaller cell group in the POA (Franceschini et al., 2006; Smith et al., 2007; Wakabayashi et al., 2010). The present findings suggest the bovine expression of kisspeptin is more closely aligned with sheep, humans, and non-human primates, whereby kisspeptin-ir and KISS1 mRNA-expressing cells are primarily localized to the ARC (Ramaswamy et al., 2008), which is an area thought to be important for both positive and negative regulation of GnRH in these species (Knobil et al., 1980; Krey, Butler & Knobil, 1975). As stated, only two animals (one DE and one PE) had identifiable kisspeptin-ir soma in the POA, those few cells were in the periventricular region of the POA. We cannot fully explain why only these two animals were positive for kisspeptin-or in this area. These animals did not have more or less kisspeptin-ir soma or fibers throughout the rest of the hypothalamus in their respective groups, but these findings suggest that kisspeptin expression in the POA of bovine is overall very low and not regulated by progesterone alone. Additional work, perhaps with colchicine pretreatment and/or *in situ* hybridization, is needed to determine the extent of kisspeptin expression in this species

The location of kisspeptin-ir cells in the bovine ARC is ideally placed to act as the interneuronal link connecting levels of sex steroids to GnRH feedback regulation. Kisspeptin expression and immunoreactivity have repeatedly been shown to be altered by gonadal steroid fluctuations. Most of kisspeptin cells contain gonadal hormone receptors and are responsive to changes in steroid levels (Adachi et al., 2007; Clarkson et al., 2008; Franceschini et al., 2006; Smith et al., 2007). In the rat, ARC KiSS-1 mRNA expression is highest at diestrus and lowest at proestrus and is increased by ovariectomy and decreased by estrogen treatment (Adachi et al., 2007). Importantly, kisspeptin/GPR54 signaling, presumably in the POA, is essential for the LH surge in mice (Clarkson et al., 2008). In sheep, a clear species difference is apparent: the MBH region of the brain, not the POA, is critical for the acute positive feedback effects of estradiol on GnRH secretion (Blache, Fabre-Nys & Venier, 1991; Caraty et al., 1998). The same may be true in the bovine. Like the bovine, ARC kisspeptin-ir neurons in the goat, sheep, and doe are more abundant during the follicular phase (low progesterone) compared to the luteal phase (high progesterone) (Jafarzadeh Shirazi et al., 2014; Smith et al., 2009). Similarly, the mean number of kisspeptin-ir cells in the doe ARC was similar during the luteal phase and anestrus, suggesting kisspeptin expression in the luteal animals was already depressed (Wagner et al., 2008). Likewise, after P4 treatment of ovariectomized animals, and as progesterone levels rise during pregnancy, cells expressing KiSS1 mRNA in the sheep ARC decrease (Sabet Sarvestani et al., 2014; Smith et al., 2007). Interestingly, our data further suggests that progesterone alone or progesterone with an unchanging level of estradiol can decrease kisspeptin levels in the ARC. The present results indicate that distribution of kisspeptin-ir cells and changes across the estrous cycle in the female bovine are similar to that in the ewe, doe, goat, and female rhesus monkey.

It has been shown that kisspeptin neurons are located upstream of GnRH neurons to stimulate LH release (Seminara, 2005). Because the excitatory effect of kisspeptin on gonadotropin secretion is inhibited by GnRH antagonists (Navarro et al., 2005), and as kisspeptin administration to hypothalamo-pituitary disconnected ewe models could not change LH concentration (Smith et al., 2008b), it has been concluded that kisspeptin acts at the hypothalamic level, not the pituitary, to stimulate GnRH release. Kisspeptin-ir contacts have been observed on GnRH cell bodies and dendrites in mice (Clarkson & Herbison, 2006), sheep (Smith et al., 2008a), horses (Magee et al., 2009), monkeys (Ramaswamy et al., 2008), and humans (Hrabovszky et al., 2010). In sheep, these contacts co-localize with synaptophysin, providing further evidence of functioning synaptic terminals (Smith, 2008). Along with fiber-somal connections, kisspeptin-ir fibers contact GnRH fibers in the ME of the mouse (Uenoyama et al., 2011) and goat (Matsuyama et al., 2011). GnRH fibers in the ME have often been implicated in control of GnRH pulsatile release (d’Anglemont de Tassigny et al., 2008; Keen et al., 2008). The presence of kisspeptin-ir fibers in the ME, alongside GnRH-ir positive fibers, suggests similar regulation is occurring in the bovine.

In conclusion, more studies are needed to determine the precise role RFRP-3, kisspeptin, and dynorphin play in the neuroendocrine control of bovine reproduction. However, it is clear from these results that the neuroanatomical distribution, possible synaptic connectivity, and response to altered progesterone levels suggest these neuropeptides play a pivotal role in the regulation of the reproductive cycle in cattle.

## Supplemental Information

10.7717/peerj.1833/supp-1 Supplemental Information 1 Raw Date Tanco et al-PeerJ Each tab is labeled with fig for which data was used.

## Additional Information and Declarations

### Data Availability

The following information was supplied regarding data availability: The raw data has been supplied as Supplemental Information 1.
